# Editorial Department

**Published:** 1880-04

**Authors:** 


### EDITORIAL DEPARTMENT.

WITH regret we announce that Edward C. Spitzka, M. D., under whose editorship THE ARCHIVES sprang into existence, has now retired from its further editorial control. This will, no doubt, be a matter of disappointment to the readers and patrons of this journal; but Dr. Spitzka's engagements in other departments of scientific research have now become so numerous as to occupy his whole time and attention, and thus make it impossible for him to attend to the management of this periodical. The printing and publishing of THE ARCHIVES will still remain in the hands of W. L. Hyde & Co.

---

WE received, a few days ago, from Albany, a copy of a bill introduced into the Assembly of this State by Mr. Travis, and which, by last advices, had been reported favorably to the Committee of the Whole, where we presume it still now rests. It is entitled "An Act to Regulate the Practice of Veterinary Medicine and Surgery in this State." We have rarely seen brought before our legislature a bill relating to any practical subject which contains a greater number of objectionable features than the one in question. In fact, it hardly possesses a single redeeming feature.

Section 1 requires the Governor of the State to appoint annually a board of examiners and censors in veterinary medicine and surgery. Taking it for granted that any such legislation is necessary to regulate this species of practice—which we are by no means prepared to allow—must it not occur to every right-minded man, interested in the general welfare of sick animals, that liability to such frequent change in the membership of the said board will have a pernicious effect upon the future status of this medical and surgical specialty. Our governors are human, and usually politicians; hence the making up of this board would soon, if not at once, come to be regarded as among the spoils of office. Again, physicians and surgeons who are not graduates, but who are merely the licentiates of, it may be, some unimportant county medical society, and practitioners of low professional standing, would, should this bill pass, become eligible to at least two places on the board.

Section 2 requires each member so appointed to hasten to put on file the notice of his appointment, together with his acceptance thereof; but it does not require of him any guarantee for the faithful performance of his official duties.

Section 3 directs that the board shall meet for business at least three times a year, at such places and times as it shall appoint. The requirements of this section would be fully complied with should the board meet three times in the same place, and should it hold three sessions all on the same day.

Section 4 is calculated to have a discouraging effect upon the veterinary colleges, which are now making vigorous efforts to advance the study and knowledge of this branch of medical practice.

We will not pursue the review of the bill further, but will simply say that its each and every section has its weakness—weak, even, as the fee section—which we are sure is too weak for the most hungry politician in the most barren district in the State. We here give this section (section 5) in full: "Every candidate, prior to his examination as above provided, shall pay to said board the sum of five dollars, to be equally divided among the members of such board, who shall receive no other or further compensation for their services."

Were we to suggest for the consideration of our honorable legislators any bill having for its object the regulation of the practice of veterinary medicine and surgery, it should be one making all laws which apply to the practice of human medicine and surgery apply with equal force to veterinary practice. This would shorten the business considerably, save a deal of trouble, and much paper and ink. But we have no faith in the efficacy or even practicability of legislation for the regulation of any such matters. Educate the people we say, and quacks, and even regulars not possessed of the necessary qualifications, will leave the field, fleeing like night hawks before the rising sun. Laws to prevent violence and to punish the transgressor, and the like, are highly necessary and even indispensable; but laws seeking to prevent the individual from using upon his own person or property any remedial agencies he may see fit to employ, will surely fail, and should fail, in the future, as they have in the past. Every physician and surgeon in the city of New York knows what pitiable failures have been all legislative enactments having in view the protection of the practitioners of human medicine; or, as some would say, the protection of the community against fraud. Yea, they have been even worse than failures; for they have served to press into strong and well organized bodies the practitioners of almost every form of senseless quackery, and thus enable them to take refuge in strongholds that cannot be successfully assailed. The New York County Medical Society practically bears witness to this, at it has recently been obliged to admit to membership persons coming from irregular schools which have been outgrowths from these organized bodies of quacks. This humiliation has been clearly effected by the stringent legislation referred to.

---

Veterinarians will, no doubt, view with regret the lengthy criticism from the pen of John Henry Steele, an English veterinary surgeon and editor of the *Veterinarian*, on the status of veterinary science on this side of the Atlantic. It can hardly be said that any of his points are well taken, and some of them are frivolous. He criticises the Columbia Veterinary College and School of Comparative Medicine of New York City in a way which a man of his professional standing would not do did he know more of it. We hope that the gentleman may have an opportunity to visit this country and the Columbia Veterinary College, and have his mind disabused of any such narrow and illiberal views.

In the meantime we would recommend that Mr. Steele dismiss the gentleman who at present occupies the position of foreign correspondent in New York, and secure another who is not so partisan, and who will take into consideration scientific attainment and other meritorious qualifications rather than the windows in the college building. The criticism also alludes, in somewhat sneering terms, to the recent graduation of the Demonstrator of Anatomy in the Columbia Veterinary College. We would here state that this gentleman was appointed at the instance of Dr. Spitzka, the late editor of this journal, whose assistant he had been, and of whose fitness for the position Dr. S. was, therefore, well qualified to judge.

---

We notice that there is now under discussion in Congress a bill having for its object the prevention of contagious disease among cattle throughout the United States and Territories. We earnestly hope that this bill will pass in some such form as will make its provision practical in the application. We think that some such quarantine regulations, such as at present exist regarding persons, should be established in regard to the entry at our ports of cattle and other kinds of live stock, and that the general government will appoint a well qualified commission with power and means necessary to stamp out such destructive contagious diseases as may at present exist among the flocks and herds of our country.

---

### COMMENCEMENT, COLUMBIA VETERINARY COLLEGE.

The commencement exercises of the above institution took place at Chickering Hall, Friday evening, March 26th. The auditorium was well filled with students, graduates, and friends of the college. The audience, a large number of whom were ladies, manifested great interest during the entire proceedings. The stage, which was occupied by the faculty, trustees, and invited guests, was elaborately decorated with beautiful floral designs and rare exotic plants. After an introductory overture by the orchestra, the names of the following gentleman were announced as having successfully passed the junior examination:

Mark L. Fry, Eli Judson Peck, William Soula, John Campbell Wallace, Emil N. Suvale, John H. Dancer, Louis Marks, Flavius Josephus Smith.

With a few appropriate remarks President Hadden presented each gentleman with a Junior Certificate, as evidence of his proficiency. The degree of Doctor of Veterinary Science was then conferred on John Lindsay, Long Island; Nathaniel Foote Thompson, N. Y. City; John Alex. McLaughlin, Jr., New Jersey; Charles D. House, New York. Honorary Degrees were conferred on Prof. Manly Miles and C. L. Hardin.

The awarding of the prizes was awaited with undisguised anxiety by the competitors and their friends; the names of the successful candidates being kept a profound secret until the owners were summoned on the platform to receive their reward from the hands of Prof. Hawkins.

The gold medal, awarded to the senior student taking highest rank on the general final examination, was bestowed on John Lindsay.

Honorable mention was made of John A. McLaughlin, Jr.

The silver medal, designed for the student of the Junior Class showing highest proficiency on the final written examination, was awarded to Mark L. Fry.

In this connection honorable mention was made of John H. Dancer and John C. Wallace.

A valuable silver medal, presented by Prof. James Hamill to the student passing the best examination in the art of scientific shoeing, was awarded to John A. McLaughlin, Jr.

The anatomical prize, a case of instruments, offered to the student preparing the best anatomical specimen for the College Museum, was awarded to N. F. Thompson.

The jurisprudence prize was carried off by John Lindsay.

The Valedictory, which was of more than usual merit, and very ably delivered, was pronounced by Nathaniel Foote Thompson.

The exercises closed with an address from Prof. Fordyce Barker, of Bellevue Medical College, and an oration by Congressman Dr. Geo. B. Loring, of Massachusetts.

---

Owing to the shortness of time before going to press, we are compelled to dispense with even a brief report of the addresses, but they will appear in full in a future number.

---

Dr. Spitzka in his withdrawal from the editorship of this periodical expresses the hope that the same encouragement which has been kindly extended to himself be extended also to his successor.

---

### ANTHRAX AND THE GERM-THEORY OF DISEASE.

---

The distinguished president of the *Societe de Biologie*, M. Bert, suggests that there are two entirely distinct infectious elements in the blood of cattle perishing from anthrax ; one, which is responsible for the virulent effects, is of a *chemical* nature, and is precipitated by alcohol ; the other consists in the bacteria, and produces merely a local affection ; it may, however, prove fatal. From his experiments, he concludes that the bacteria are neither the essential nor the necessary results of the disease, as there may be few bacteria, and an intense constitutional affection on the one hand, and many bacteria, but a less intense disease on the other. He is necessarily forced to the conclusion that two different affections are confounded as anthrax. The one is the genuine affection which is analogous in its virus to glanders and variola ; the other is micro-parasitic. Both conditions may, and frequently do co-exist, we presume because the "chemical" poison produces such a state of the blood as to render it a favorable soil for the developement of *micrococci* and *bacilli.* The researches of Mr. Bert are to be considered as an important feature of the reaction which is now taking place against the over-strained germ-theory of disease. We have no doubt that a careful comparison of the results obtained by different observers will show that molecular movements and microcytes have frequently formed the basis for new "germ-theories" in some cases, and the accidental presence of bacteria, such as are found even normally in the oral mucus, in others (diphtheria). A judicious study of the finer chemistry of the fluids in infectious diseases would probably lead to more definite and more valuable results. The very remarkable discovery of Bollinger, that in the *Emphysema contagiosum* of the Bavarian cattle, marsh gas was produced in the subentaneous tissues as well as in the *living* blood and lymph, and the resistance which animals of certain species show to certain infectious diseases, are suggestive in this direction. Those provided with an acute sense of smell are well able to distinguish between the odors of the blood and parenchymatous fluids of the horse and cow, the cat and dog, man and the chick. We have been able to detect a specific odor of these fluids in many wild animals.* These differences in odor probably depend on subtle chemical differences, which may exert an influence on the reception of the more or less chemically complex body which constitutes the virus. It is remarkable in this connection that the specific odor of small-pox, scarlatina, measles (like freshly picked geese), and other diseases, are analogous to animal odors, or even can be identified with these.

---

* The bear and baboon have an odor which is a modification of that of the dog; the turtle, alligator, iguana, and snake can be classed together and resemble another sauropsidean, the bird. The frog, toad, and salamander, as well as the proteus, differ from the above, but resemble each other. The deer is more like the cow, the hippopotamus and elephant like the horse, the paca like the rabit, &c.

